# Supplementary material for: DNA polymerase κ-dependent DNA synthesis at stalled replication forks is important for CHK1 activation
Source: EMBO J. 2013 Jun 25;32(15):2172–85. doi: 10.1038/emboj.2013.148 (PMC3730229; doi:10.1038/emboj.2013.148)

**Supplemental material: additional Materials and Methods and 5 supplemental figures**

## *Additional Materials and Methods*

*Pol κ -/- MEF*

The embryonic fibroblasts (MEF) used in this study were generated from homozygous mutant Pol κ-deficient mice described in (Schenten et al, 2002)

*Preparation of polyclonal anti-rabbit IgG antibody of human Pol κ*

The human POL K gene (GenBank accession number XM_003930.2), was amplified by polymerase chain reaction (PCR) from human testis large insert cDNA library (CLONTECH). The amplified fragments were digested with *Nco*I and *Bam*HI and the resulting fragment was ligated into similarly digested vector pYG8582, which is the same as pET-16b (Novagen) but has the translational DB enhancer. The resulting plasmid pYG8583 codes for an N-terminal 10xHisTagged full-length Pol κ. To construct the C-terminally truncated Pol κ expression vector, a synthetic linker was ligated between the *Xba*I and *Bam*HI sites of pYG8582. This construct was then digested with *Xba*I and *Avr*II, and the digested plasmid was ligated with the *Xba*I fragment of pYG8583 carrying the N-terminal portion of the Pol κ coding sequence. The resulting construct overexpressing C-terminally truncated 10xHis Tagged Pol $\kappa_{1\text{-}559}$ has been named pYG8591. To express the truncated 10xHis Tagged Pol $\kappa_{1\text{-}559}$ plasmid pYG8591 was transformed into *E. coli* Rosetta competent cells (Novagen) and the expression was induced by adding IPTG. Cells were harvested and resuspended in BugBuster lysis buffer (Novagen) and soluble proteins were collected by centrifugation. To purify recombinant Pol κ, the protein was bound to BD TALON

Superflow resin (BD Biosciences) and eluted according to the manual provided by BD Biosciences. The eluted proteins were further purified by gel filtration, followed by ion exchange chromatography (HiTrap Heparin HP, GE healthcare) using the FPLC system (AKTAexplorer 10s, GE healthcare). The polyclonal anti-rabbit IgG antibody of Pol κ was obtained by injection of the purified protein to rabbits (Takara, Japan).

*Antibodies for Immunoblotting*

Experiments with Xenopus extracts. Anti-Xpol κ antibodies were produced against full-length Xenopus Pol κ made in bacteria as 6His-tag recombinant protein as previously described (Yagi et al, 2005) at the external facility Proteogenix (Strasbourg, France). Inclusion bodies were isolated and the protein purified on a nickel column followed by step dialysis renaturation and injection into rabbits. Anti-RPA antibodies were produced as described (Recolin et al, 2012). Anti-MCM3 antibodies have been previously described (Coue et al, 1998). Histone H3 and DNA polymerase α (p180) antibodies were purchased from Abcam (ab1791 and ab31777 respectively). Anti-hChk1 P-S345 antibody was from Cell Signaling (2341, recognizes P-S344 in Xenopus), anti-hChk1 antibody from Santa Cruz (sc-8408). Anti-RAD9 and ATR antibodies were described in (Recolin et al, 2012). XTopBP1 antibodies were produced as previously described (Parrilla-Castellar & Karnitz, 2003). Anti-PCNA antibody used to detect XPCNA was from Sigma (clone PC10). ORC2 antibody was produced against 6His-tagged bacterial recombinant proteins (a gift of Marcel Méchali, IGHG-CNRS Montpellier). Anti pol δ antibodies were previously described (Van, et al., 2010). Hybridization of antibodies to nitrocellulose membranes was performed using a SNAPi.d.® system (Millipore) and detection was performed by Enhanced Chemio Luminescence (Luminata Crescendo® reagent, Millipore).

Experiments with mammalian samples. Rabbit polyclonal antibodies: Actin (Sigma, 1:30000); MCM7 (Santa Cruz, WB, 1:1000), phospho-Chk1-Ser345 (Cell signaling, 1:1000), Pol η (Abcam, 1:1000), Pol α (Abcam, 1:1000). Rat antibody: CDC45 (a kind gift from Heinz-Peter Nasheuer, Galway-Ireland; 1:50). Mouse monoclonal antibodies: Actinin (Chemicon, 1:2000); Chk1 (Santa Cruz, WB, 1:1000); MCM2 (Abcam, 1:3000); RPA-34 (Calbiochem, 1:1000); Tubulin-alpha (Sigma, 1:50000); ORC4 (1:1000); PCNA (Abcam, 1:1000). Secondary antibodies: HRP conjugated anti-rabbit and HRP conjugated anti-mouse (Jackson Immuno Research, 1:20000). HRP conjugated anti-rat (1:5000).

*Additional antibodies (supplemental Figures)*

Rabbit polyclonal antibodies: ATR (Cell signaling, 1:1000); ATRIP (Cell signaling, 1:1000); Hus1 and Rad9A (kind gift from Pr U Hubscher, University of Zurich). Mouse monoclonal antibodies: Pol δ (Santa Cruz, 1:250).

***Supplemental figures***

**Supplemental Figure S1: Validation of the specificity of Pol κ depletion in human cells** (**A,B**) Western blot analysis of whole cell extracts prepared from Hela and MRC5 cells, untransfected (unt), transfected with control luciferase siRNA (si-luc), Pol κ individual siRNA (si-κ1 and si-κ2) or with two independent Pol κ siRNA pools (Dharmacon) (D-si-κ and si-κ3'UTR). Actin, actinin or Orc 4 were used as loading controls. (**C**) mRNA expression of several replicative and specialized DNA polymerases was analysed by quantitative real time PCR in MRC5 cells transfected with control luciferase siRNA (si-luc), or Pol κ individual siRNAs (si-κ1 and si-κ2).

Data are mean +/- SD from at least three independent experiments. The p-value determined with a t-test *** $p<0.0001$. Only the Pol κ expression is significantly decreased after si-RNA transfection. (**D**) Western blot analysis of whole cell extracts prepared from 293T untreated or treated with HU (2 mM, 3h), transfected with control luciferase siRNA (si-luc) or Pol κ individual siRNA (si-κ1). Quantification of Pchk1/Chk1 is the mean +/- SD of two independent experiments. Actin is used as a loading control. (**E**) Western blot analysis of whole cell extracts prepared from MRC5 and Hela untreated cells transfected with control luciferase siRNA (si-luc) or Pol κ siRNAs (si-κ1 and si-κ3'UTR) and analysed by immunoblotting with the indicated antibodies. Tubulin α and actinin are used as a loading control. (**F**) Whole cell extracts from control and Pol κ -/- MEF, untreated or treated with HU (1 mM, 1h) were prepared and analyzed by Western blot with the indicated antibodies. Quantification of the ratio P-CHk1/Chk1 in treated vs untreated cells is presented. (**G**) Western blot analysis of whole cell extracts prepared from MRC5 untreated or treated with HU (2 mM, 3h), transfected with empty vector (-) or vectors expressing FLAG-tagged wild-type Pol κ (Pol κ-WT) respectively. Actinin is used as a loading control.

**Supplemental Figure S2: Additional experiments confirming the phenotype of Pol κ-depleted cells** (**A**) The level of Pol κ on chromatin was analyzed by Western blotting in 293T cells, untreated or treated with 2 mM HU 3h; Tubulin α and Histone H3 were used as loading controls for chromatin and soluble fractions respectively. (**B**) MRC5 cells transfected with the indicated siRNAs were mock-irradiated or irradiated with 50 $J/m^2$, then cell extracts were fractionated, and soluble fractions were analyzed by immunoblotting with the indicated antibodies.

**Supplemental Figure S3: Functional characterization of Xpol κ** (**A**) Western blot of pre-immune (PI) or immune serum (XPol κ) of rabbits immunized with recombinant Xenopus Pol k protein. The Xenopus Pol κ serum recognizes a specific polypeptide at the expected size of 100 kDa. (**B, upper panel**) Egg supernatants mock-depleted or depleted with the XPol κ antibodies were reconstituted with sperm chromatin (2000 nuclei/µl) in the absence (-) or presence (+) of aphidicolin (15µM) or after UV irradiation (800 J/$m^2$) and incubated at room temperature for 90 minutes. Isolation of nuclei and preparation of chromatin and nuclear soluble fractions were performed as described in Materials and Methods. Proteins fractions were then analysed by western blot with the indicated antibodies; (**B, lower panel)** Analysis of Chk1 phosphorylation in Mock-depleted extracts (ΔMock) with (+) or without (-) UV irradiation (800 J/$m^2$) or aphidicolin (15 µM). (**C**) Replication fork uncoupling occurs normally after incubation of UV-irradiated sperm chromatin in Xenopous egg extracts. Mock (- UV) or UV-irradiated (+ UV) sperm chromatin was incubated in Xenopus egg extracts Mock-depleted (ΔMock) or depleted with Xpolk antibodies (ΔXpolk) for 90 minutes. Chromatin fractions were obtained as described in Materials and Methods and analyzed by western blot with the indicated antibodies. (**D**) Sperm chromatin was replicated in egg extracts mock-depleted (ΔMock) or depleted with XPol κ antibodies (ΔXPol κ) containing or not 15 µM aphidicolin (APH) and α-[$P^{32}$]dCTP. At 40 minutes, total DNA was purified as described (Van et al, 2010), replication intermediates were fractionated by denaturing polyacrylamide gel electrophoresis and detected by autoradiography after exposure to a PhosphoImager screen (Molecular Dynamics). Abundance of 25-150 nt long DNA intermediates was quantified by densitometric scanning and analysed with ImageJ software. (**E**) The abundance of Pol α and Pol α in extracts Mock-depleted (ΔMock) or XPol κ-depleted

(ΔXPol κ) was analysed by immunoblotting. (**F**) Addition of purified recombinant Pol κ to egg extracts depleted of XPol κ totally restored recruitment of Rad9 onto chromatin after aphidicolin. Sperm chromatin was incubated in XPol κ (ΔXPol κ) depleted egg extracts in the presence of aphidicolin (15 µM) for 60 min. Chromatin fractions were isolated after 90 minutes incubation and analysed by immunoblotting with the indicated antibodies. (**G**) Xpolk interacts with XRad9. Xenopus egg extracts were immunoprecipitated with XPol κ or Rad9 antibodies and analysed by western blot using anti-Rad9 or XPol κ antibodies. (**H**) Xpolk does not interact with Pol α nor Pol δ. Immunoprecipitation (IP) from egg extracts with the indicated antibodies was performed as described in materials and methods. Immunoprecipitates were blotted with antibodies against Xpol κ, XRad9, Xpol δ or Xpol α.

**Supplemental Figure S4: Increased spontaneous DNA damage following Pol κ depletion.**

**(A)** Additional recruitment of RPA is due to ssDNA accumulation at stalled forks and not to firing of new replication origins in Pol κ–deficient cells. Extracts of MRC5 cells transfected with control siRNA (si-luc) or siRNAs targeting Pol κ (si-κ1, si-κ2) were fractionated. Chromatin fractions were then subjected to immunoblotting with the indicated antibodies. Extracts from the untransfected cells treated with HU (2mM 3h) served as positive control for RPA hyperloading; MCM7 served as loading control for chromatin fraction. (**B**) Evaluation of endogenous DNA damage in S-phase: γ-H2AX foci formation (green) in the indicated cell lines was analysed by immunofluorescence in PCNA-positive nuclei (red) of control and Pol κ-deficient cells 48h after transfection with the indicated si-RNA. DNA content was visualised by DAPI coloration (blue). At least 100 cells were counted for each condition; scale bar 10µm. (**C**) Quantification of

γ-H2AX-positive cells by Flow cytometry analysis and by immunofluorescence for HeLa wild-type and $XPA^{KD}$ cell lines transfected with the indicated siRNAs under low (5%) or high (20%) oxygen conditions. The numbers of cells analysed were more than 300.

**Supplemental Figure S5: addition experimental evidence showing that 53BP1 nuclear bodies in G1 are increased following Pol κ depletion.**

(**A**) IgG/555 and IgG1/488 were controls of immuno-detection. Rabbit IgG and mouse IgG1 were used instead of 53BP1 and CycA antibodies respectively. Secondary antibodies and immuno-detection protocols are mentioned in material and methods. (**B and C**) Distribution of the number of 53BP1 foci per nucleus in cells untransfected (unt), transfected with control luciferase siRNA (si-Luc) or Pol κ individual siRNA (si-κ1 and si-κ2). Two independent experiments (EXP2 and EXP3) performed without treatment (untreated) or after 0.2 µM aphidicolin for 24h were showed (n=100 for each condition). Box, 25-75 percentile range; whiskers, minimum and maximum values. Mann-Whitney test was applied to compare Pol κ-deficient cells data set with control cells.

## Supplemental References

Coue M, Amariglio F, Maiorano D, Bocquet S, Mechali M (1998) Evidence for different MCM subcomplexes with differential binding to chromatin in Xenopus. *Exp Cell Res* **245:** 282-289

Parrilla-Castellar ER, Karnitz LM (2003) Cut5 is required for the binding of Atr and DNA polymerase alpha to genotoxin-damaged chromatin. *J Biol Chem* **278:** 45507-45511

Recolin B, Van Der Laan S, Maiorano D (2012) Role of replication protein A as sensor in activation of the S-phase checkpoint in Xenopus egg extracts. *Nucleic Acids Res* **40:** 3431-3442

Schenten D, Gerlach VL, Guo C, Velasco-Miguel S, Hladik CL, White CL, Friedberg EC, Rajewsky K, Esposito G (2002) DNA polymerase kappa deficiency does not affect somatic hypermutation in mice. *Eur J Immunol* **32:** 3152-3160

Van C, Yan S, Michael WM, Waga S, Cimprich KA (2010) Continued primer synthesis at stalled replication forks contributes to checkpoint activation. *J Cell Biol* **189:** 233-246

Yagi Y, Ogawara D, Iwai S, Hanaoka F, Akiyama M, Maki H (2005) DNA polymerases eta and kappa are responsible for error-free translesion DNA synthesis activity over a cis-syn thymine dimer in Xenopus laevis oocyte extracts. *DNA Repair (Amst)* **4:** 1252-1269

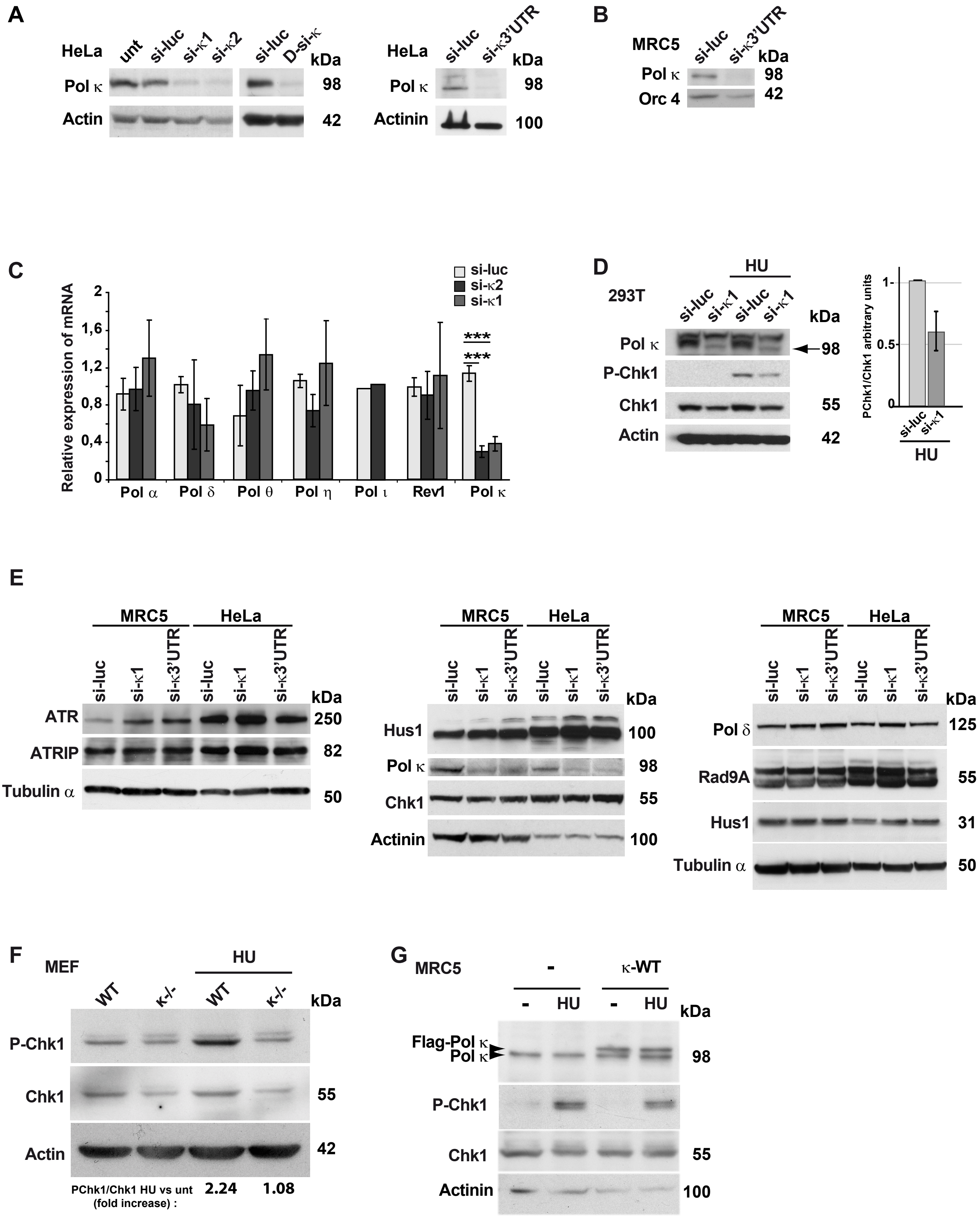
A
HeLa
unt
si-luc
si-κ1
si-κ2
si-luc
D-si-κ
kDa
Pol κ
98
Actin
42
HeLa
si-luc
si-κ3'UTR
kDa
Pol κ
98
Actinin
100
B
MRC5
si-luc
si-κ3'UTR
kDa
Pol κ
98
Orc 4
42
C
si-luc
si-κ2
si-κ1
Relative expression of mRNA
2
1,6
1,2
0,8
0,4
0
***
***
Pol α
Pol δ
Pol θ
Pol η
Pol ι
Rev1
Pol κ
D
293T
HU
si-luc
si-κ1
si-luc
si-κ1
kDa
Pol κ
98
P-Chk1
Chk1
55
Actin
42
PChk1/Chk1 arbitrary units
1
0.5
0
si-luc
si-κ1
HU
E
MRC5
HeLa
si-luc
si-κ1
si-κ3'UTR
kDa
ATR
250
ATRIP
82
Tubulin α
50
Hus1
100
Pol κ
98
Chk1
55
Actinin
100
Pol δ
125
Rad9A
55
Hus1
31
Tubulin α
50
F
MEF
HU
WT
κ-/-
WT
κ-/-
kDa
P-Chk1
Chk1
55
Actin
42
PChk1/Chk1 HU vs unt
(fold increase) :
2.24
1.08
G
MRC5
-
κ-WT
-
HU
-
HU
kDa
Flag-Pol κ
Pol κ
98
P-Chk1
Chk1
55
Actinin
100

**Bétous_Supplementary Fig 2**

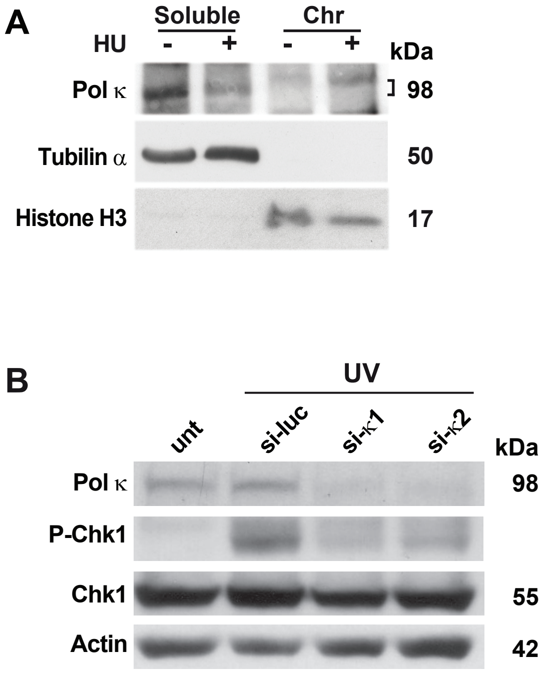

Bétous_Supplementary Fig 3

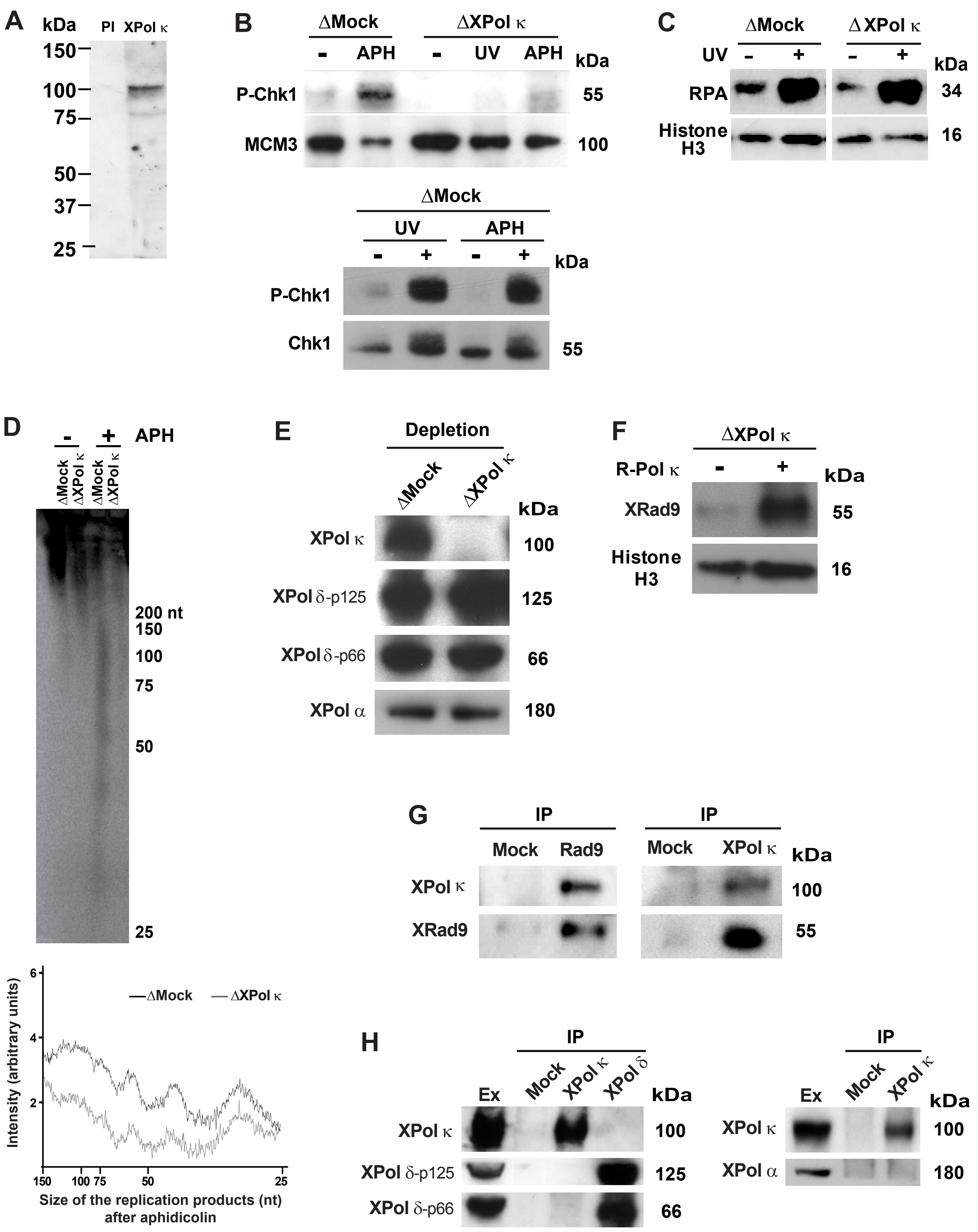

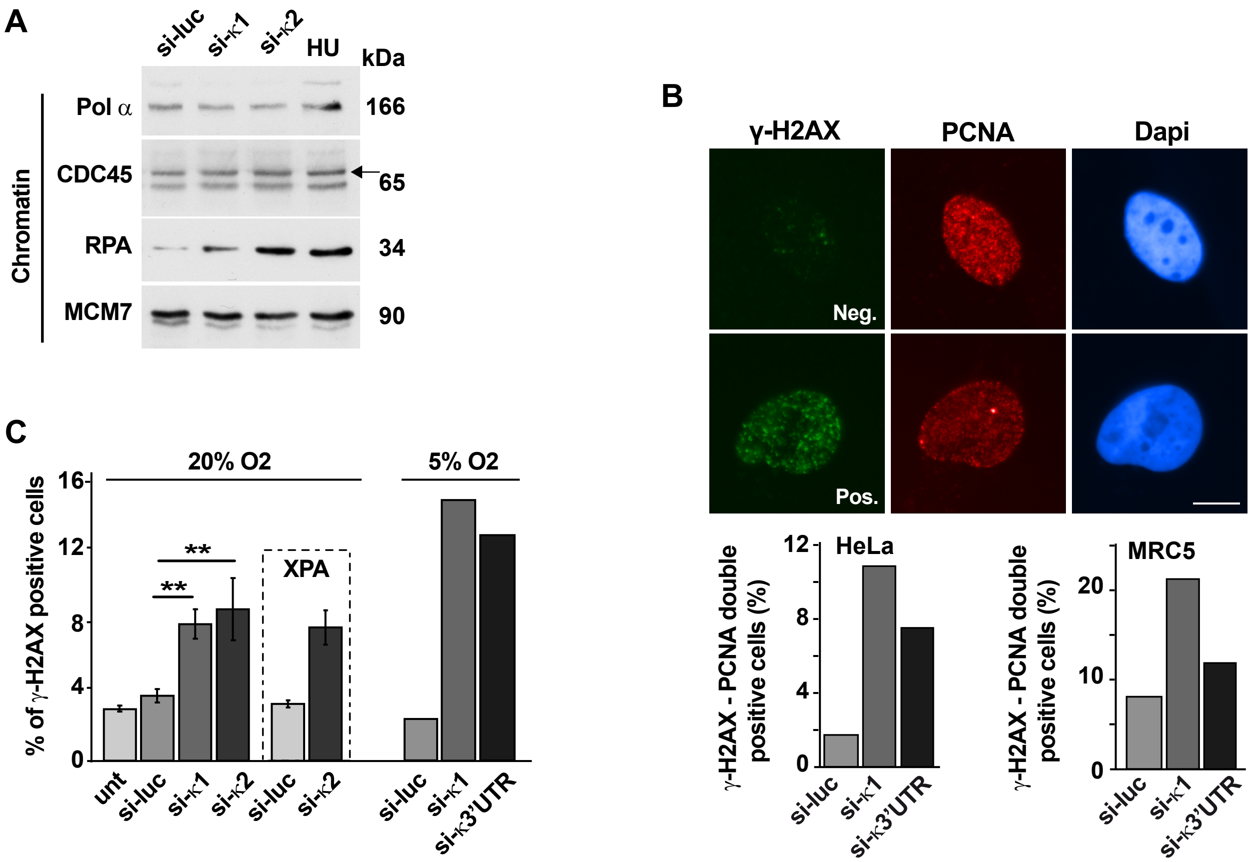
A
si-luc
si-κ1
si-κ2
HU
kDa
Chromatin
Pol α
166
CDC45
65
RPA
34
MCM7
90
B
γ-H2AX
PCNA
Dapi
Neg.
Pos.
γ-H2AX - PCNA double positive cells (%)
HeLa
MRC5
si-luc
si-κ1
si-κ3'UTR
C
% of γ-H2AX positive cells
20% O2
5% O2
XPA
**
**
unt
si-luc
si-κ1
si-κ2
si-luc
si-κ2
si-luc
si-κ1
si-κ3'UTR

A

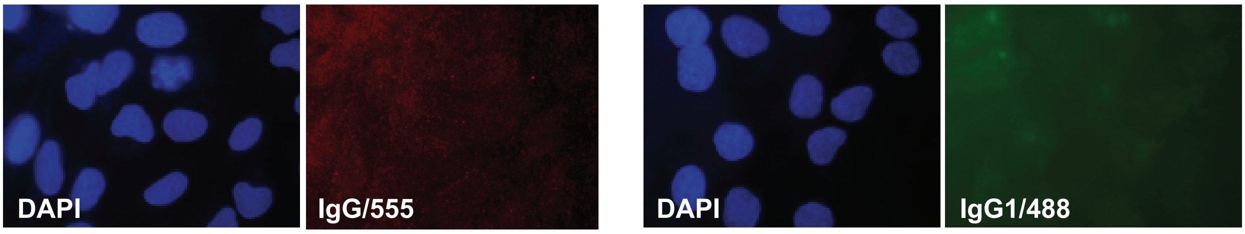


B

Untreated

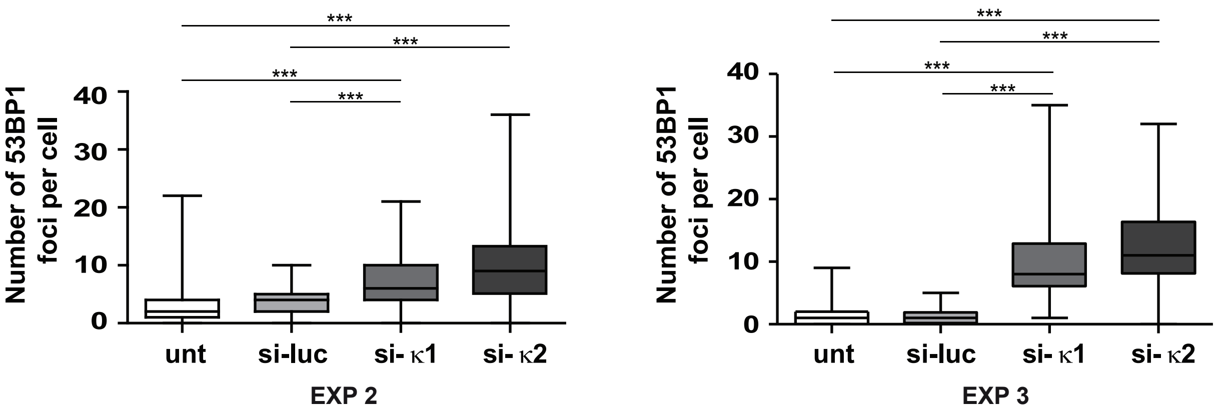


C

0.2µM Aphidicolin

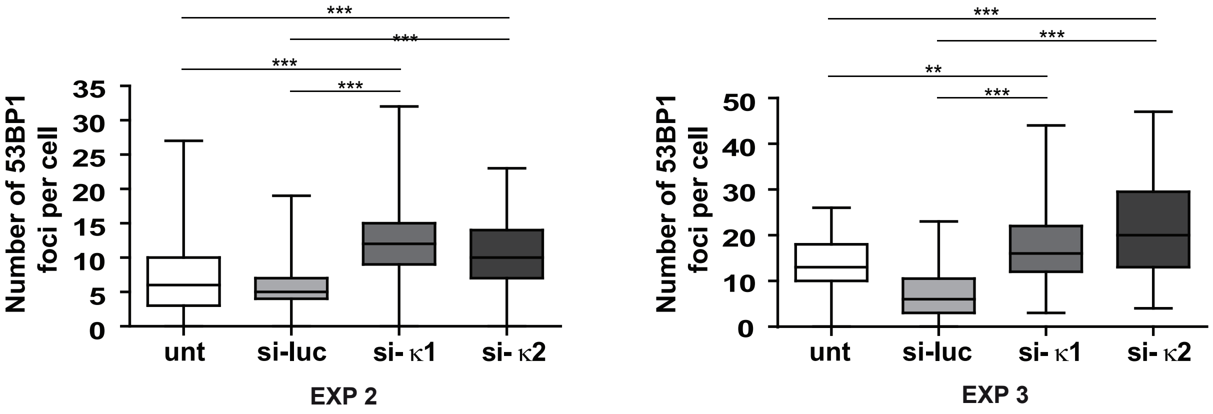

Supplement: Supplemental Information [file emboj2013148s1.pdf]
