# Supplementary figures and images for: DNA polymerase κ-dependent DNA synthesis at stalled replication forks is important for CHK1 activation
Source: EMBO J. 2013 Jun 25;32(15):2172–85. doi: 10.1038/emboj.2013.148 (PMC3730229; doi:10.1038/emboj.2013.148)

## Betous-supplementary Fig1

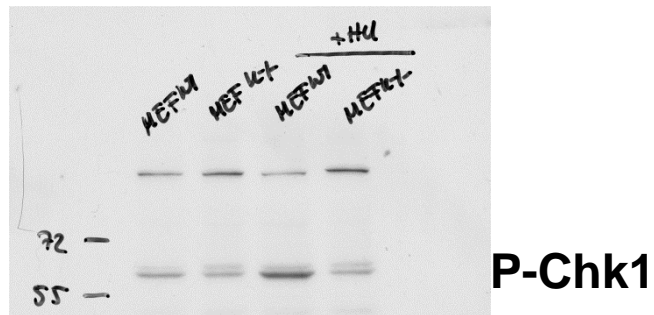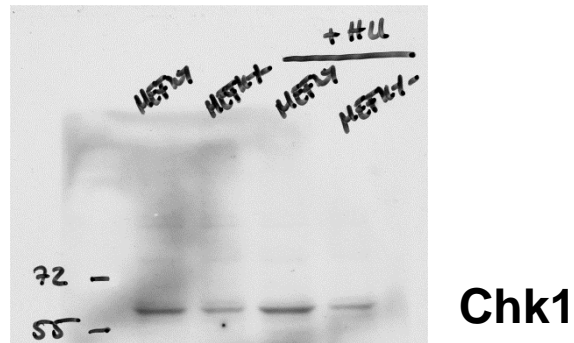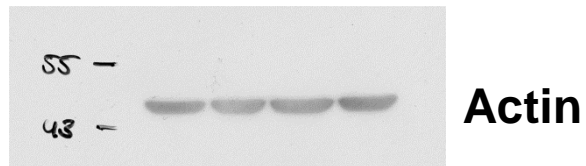

Supplement: Supplemental Information [file emboj2013148dfs1.pdf]

### Betous-Supplementary Fig 3

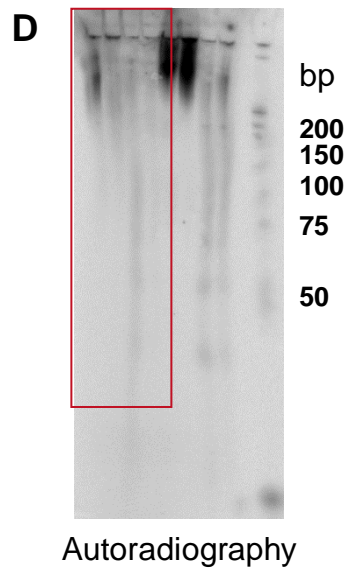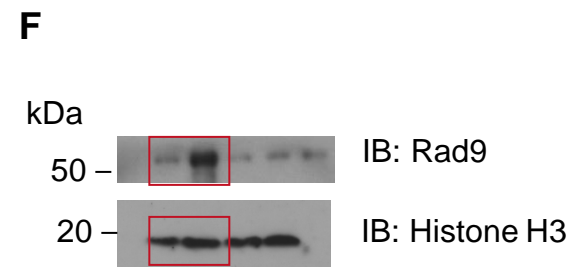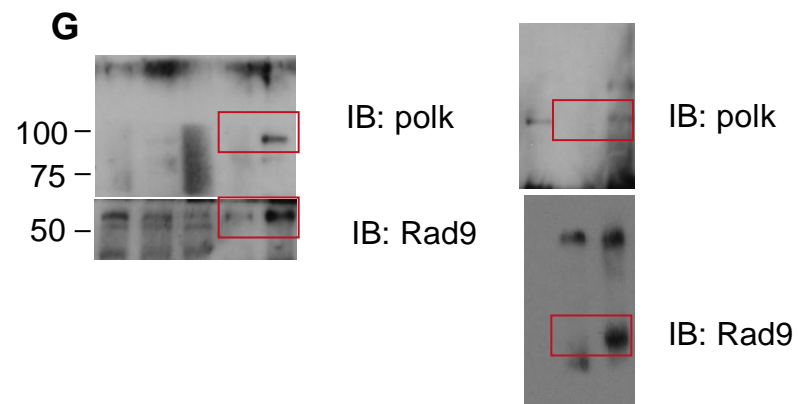

Supplement: Supplemental Information [file emboj2013148dfs3.pdf]

Betous \_Supplementary Fig 4

A

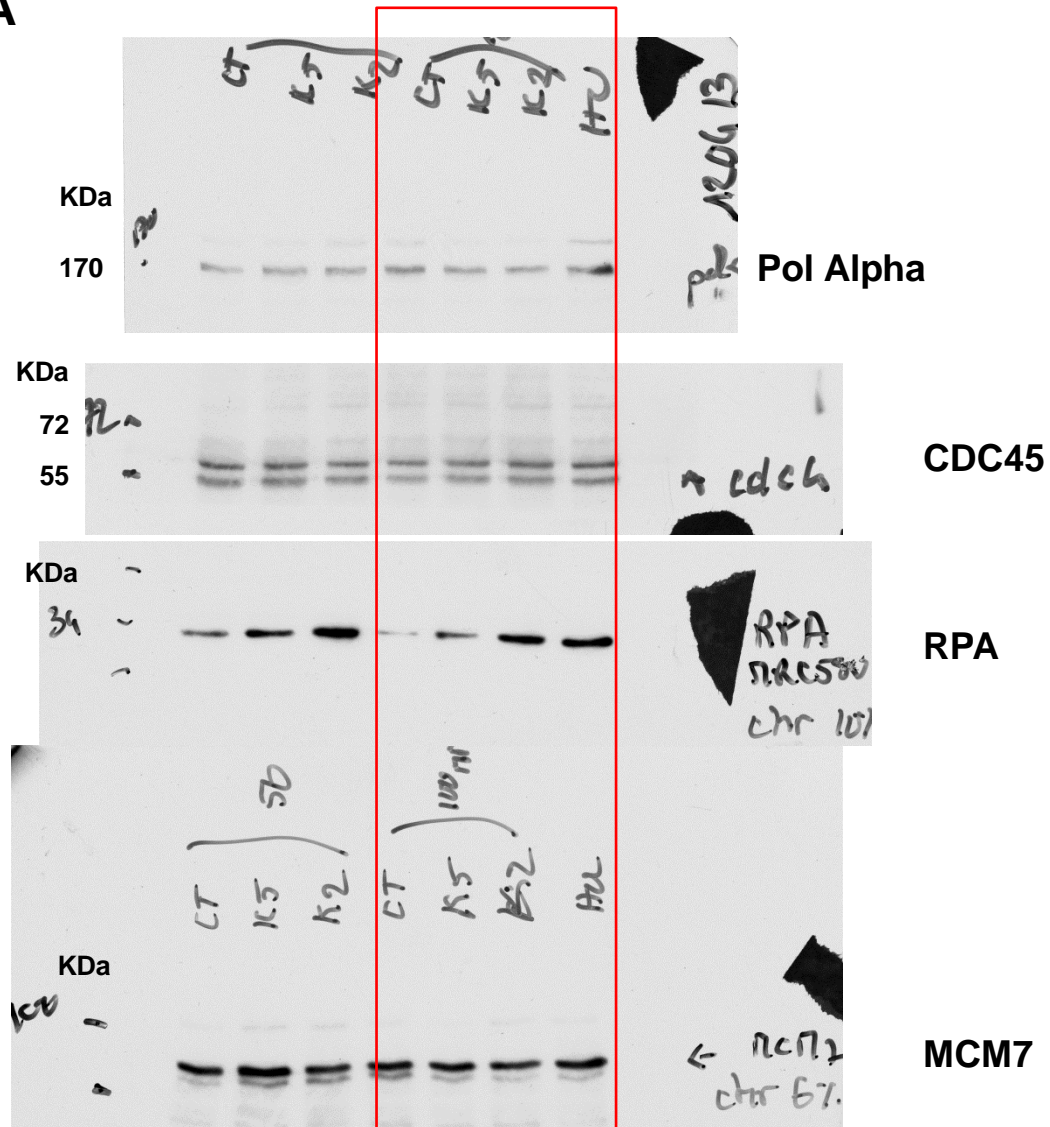

Supplement: Supplemental Information [file emboj2013148dfs4.pdf]

# Betous\_Fig1

A

Pol Kappa

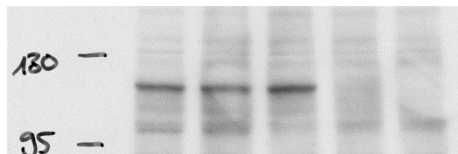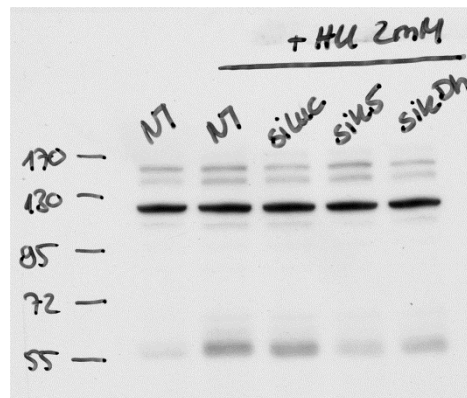

P-CHK1

Tubulin  $\alpha$

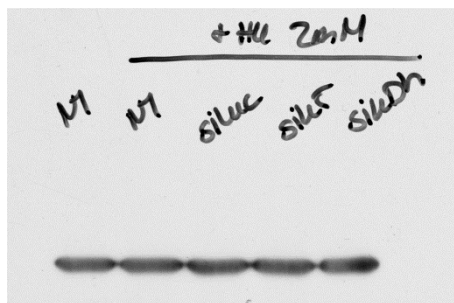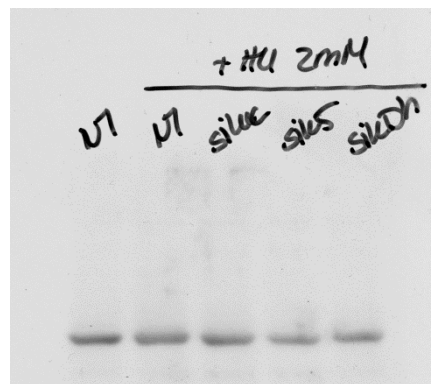

CHK1

# Betous\_Fig1

B

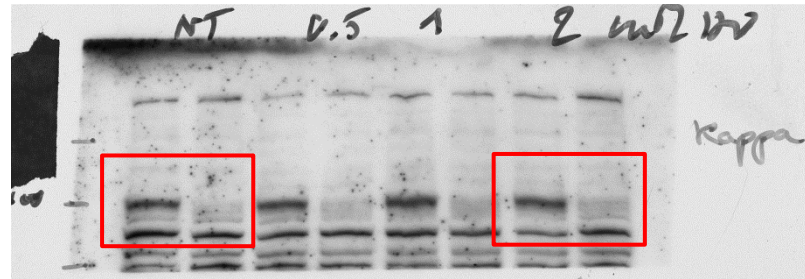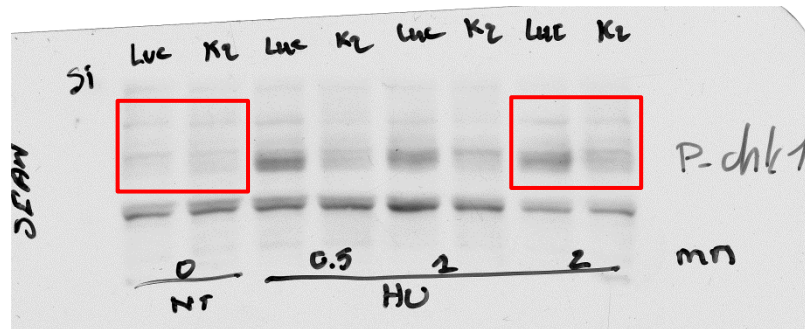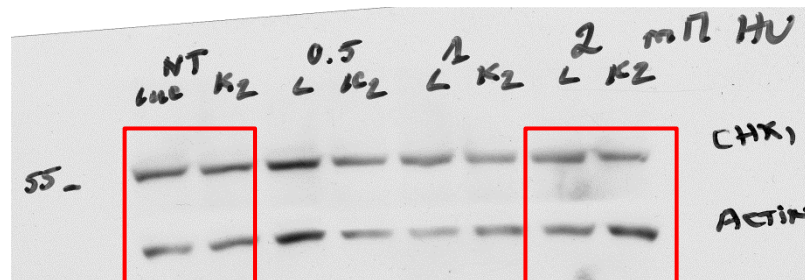

## Betous\_Fig1

**C**

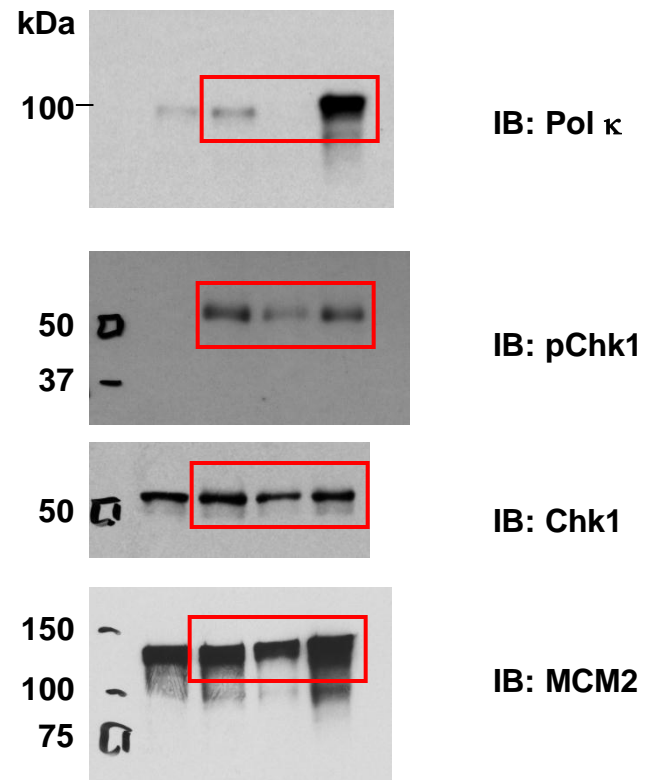

# Betous\_Fig1

D

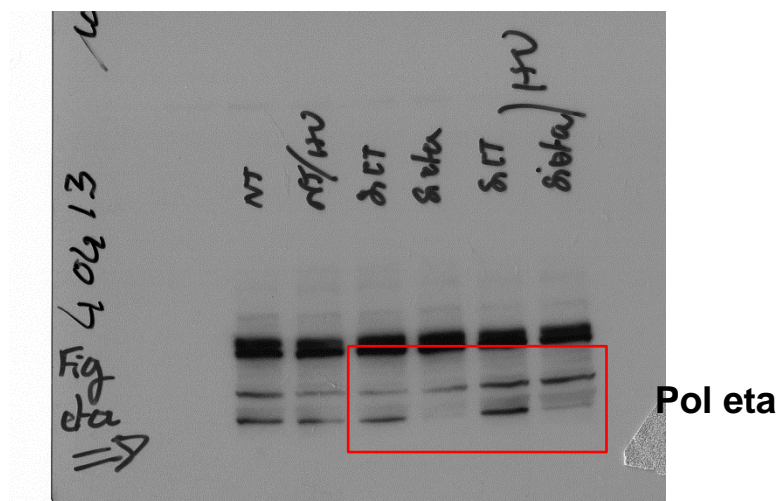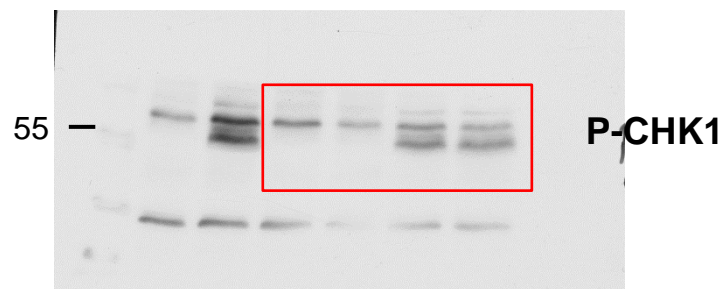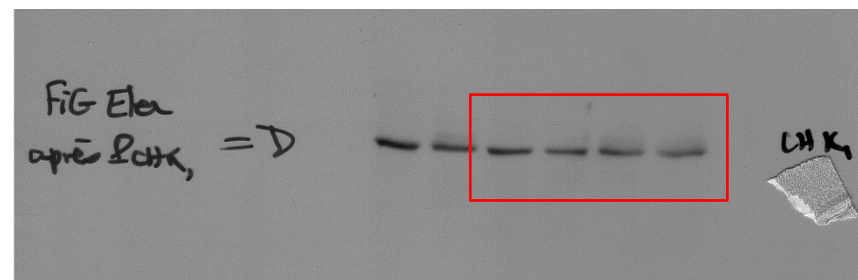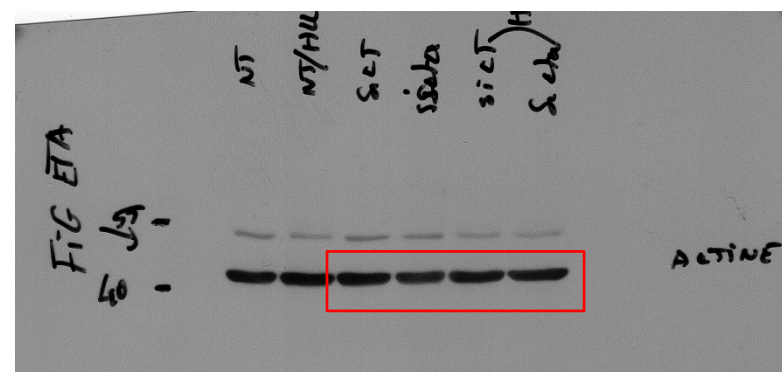

Supplement: Source Data for Figure 1 [file emboj2013148df1.pdf]

## Betous\_Fig2

**B**

**kDa**

75—

50—

37—

25—

50—

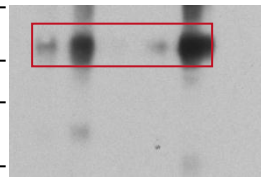

**IB: PChk1**

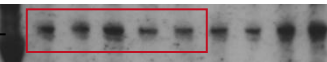

**IB: Chk1**

Supplement: Source Data for Figure 2 [file emboj2013148df2.pdf]

**Betous \_Fig 3**

**A**

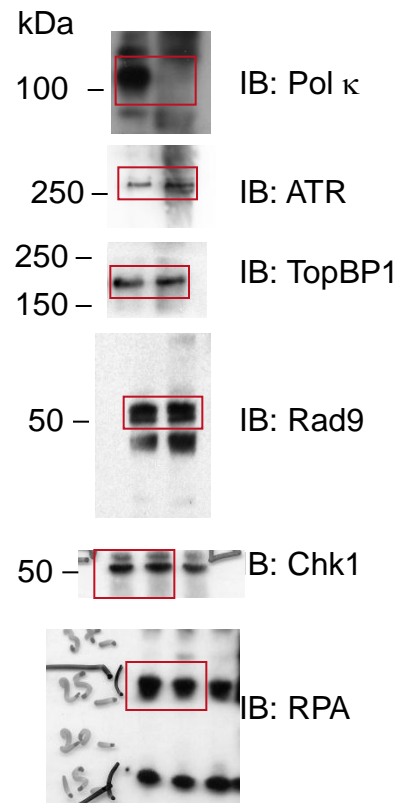

**B**

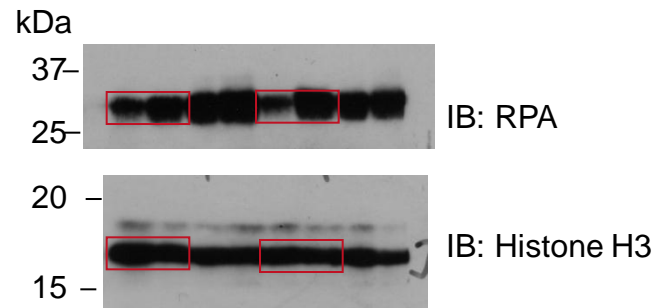

**D**

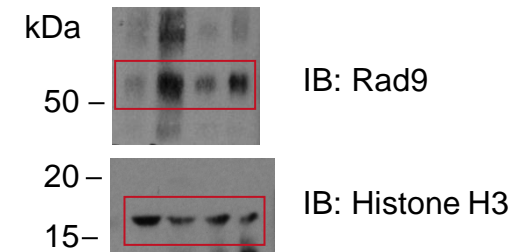

**C**

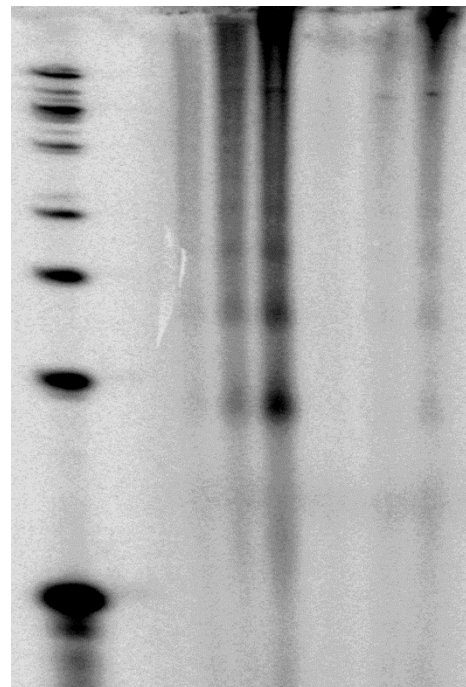

Autoradiography

**E**

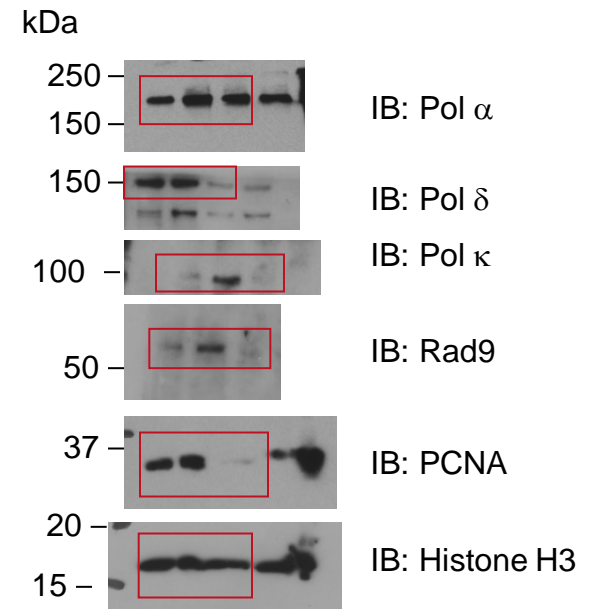

Supplement: Source Data for Figure 3 [file emboj2013148df3.pdf]

Betous \_Fig 4C

# Chromatin

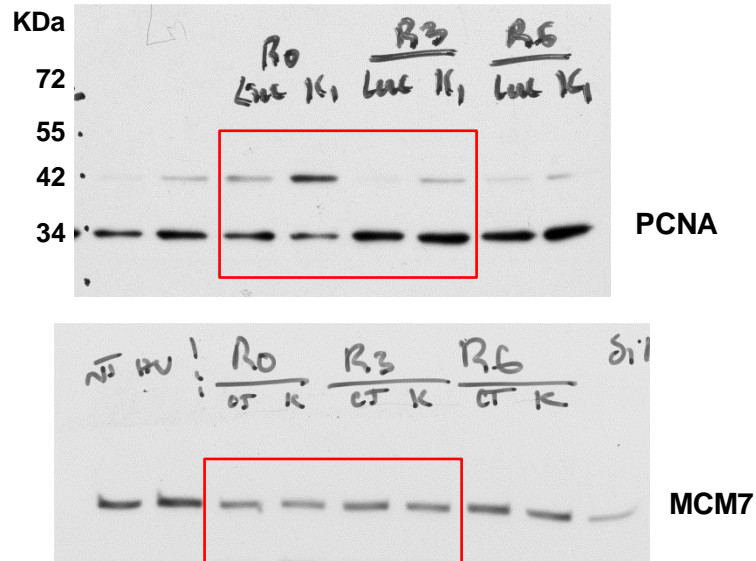

# Soluble

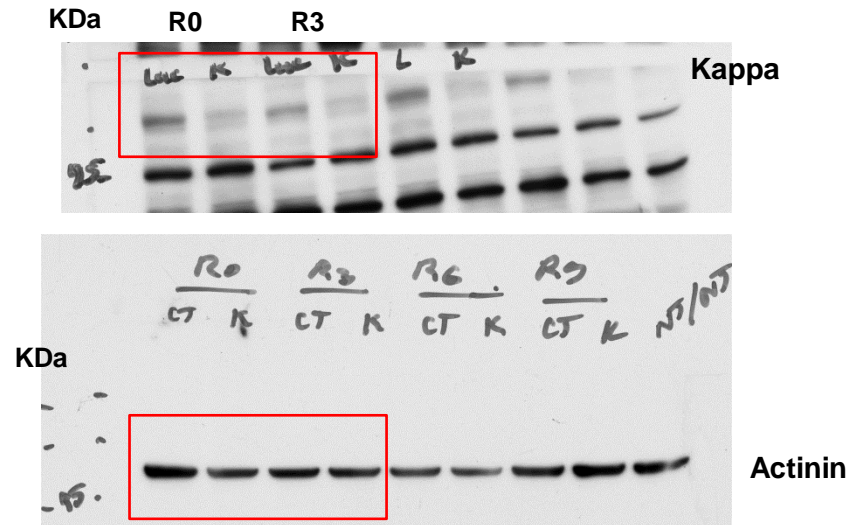

Supplement: Source Data for Figure 4 [file emboj2013148df4.pdf]

Betous\_Fig 5

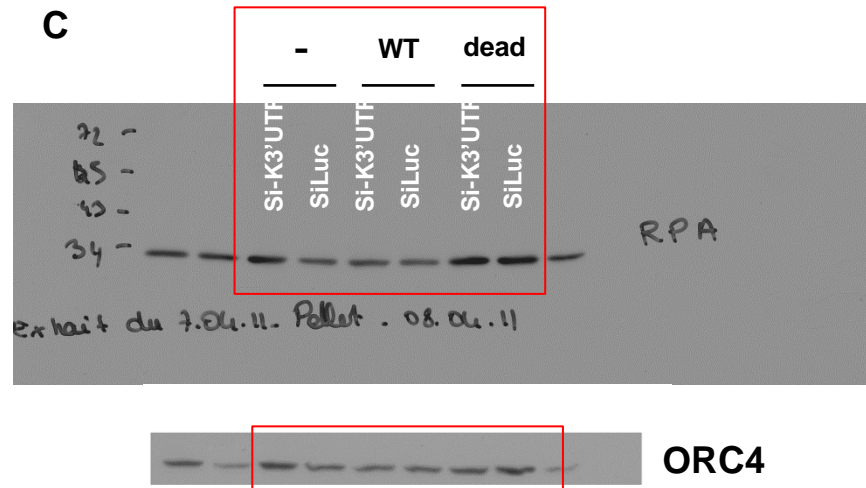

Supplement: Source Data for Figure 5 [file emboj2013148df5.pdf]
